# Leptomeningeal and Intraparenchymal Blood Barrier Disruption in a MOG-IgG-Positive Patient

**DOI:** 10.1155/2018/1365175

**Published:** 2018-12-09

**Authors:** Seyed Hamidreza Mohseni, Hanne Pernille Bro Skejoe, Jens Wuerfel, Friedemann Paul, Markus Reindl, Sven Jarius, Nasrin Asgari

**Affiliations:** ^1^Department of Radiology, Slagelse Hospital, Slagelse, Denmark; ^2^Department of Radiology, Aleris-Hamlet Hospital, Copenhagen, Denmark; ^3^Medical Image Analysis Center Basel and Department of Biomedical Engineering, University Basel, Switzerland; ^4^Clinical and Experimental Multiple Sclerosis Research Center and NeuroCure Clinical Research Center, Department of Neurology, Charité-Universitätsmedizin Berlin, Germany; ^5^Experimental and Clinical Research Center, Max Delbrueck Center for Molecular Medicine and Charité-Universitätsmedizin Berlin, Berlin, Germany; ^6^Clinical Department of Neurology, Medical University Innsbruck, Innsbruck, Austria; ^7^Molecular Neuroimmunology Group, Department of Neurology, University Hospital Heidelberg, Heidelberg, Germany; ^8^Department of Neurology, Slagelse Hospital, Institute of Regional Health Research, Denmark; ^9^Department of Neurobiology, Institute of Molecular Medicine, University of Southern Denmark, Denmark

## Abstract

**Background:**

Recently, pathogenic serum immunoglobulin G (IgG) autoantibodies to myelin oligodendrocyte glycoprotein (MOG) have been detected in a subgroup of patients with central nervous system (CNS) demyelination, including in patients with myelitis. Relatively little is known so far about leptomeningeal involvement in MOG-IgG-positive myelitis.

**Findings:**

We report the case of a 30-year-old previously healthy woman presenting with longitudinally extensive transverse myelitis and tetraparesis, in whom both the leptomeningeal barrier and the blood-brain barrier (BBB) were altered, as demonstrated by gadolinium-enhanced MRI during relapse. Blood samples taken at onset and four years later were retrospectively found positive for MOG-IgG.

**Conclusion:**

Our findings demonstrate that spinal leptomeningeal enhancement (LME) can occur in MOG-IgG-positive encephalomyelitis (EM) and may accompany intraparenchymal BBB breakdown.

## 1. Introduction

Pathogenic immunoglobulin G (IgG) autoantibodies directed to myelin oligodendrocyte glycoprotein (MOG), an oligodendrocytic protein localized to the outer surface of the myelin sheaths, have recently been identified in patients with inflammatory CNS demyelination [1–6]. This discovery has led to the recognition of a new disease entity, now often referred to as “MOG encephalomyelitis” (MOG-EM) [2]. Phenotypically, MOG-EM may present as optic neuritis (ON), (often longitudinally extensive) transverse myelitis, brainstem encephalitis, encephalitis, or any combination of these syndromes and, especially in children, as acute demyelinating encephalomyelitis (ADEM) [3–6].

Myelitis is often associated with leptomeningeal (pia-arachnoid) contrast enhancement (LME). LME is a sign of leptomeningeal barrier impairment and leakage of contrast agent into the subarachnoid space. Understanding LME patterns may help in the differential diagnosis of the various forms of myelitis [7–10]. So far, little is known about LME in MOG-IgG-positive myelitis.

## 2. Case Report

**Figure 1:**
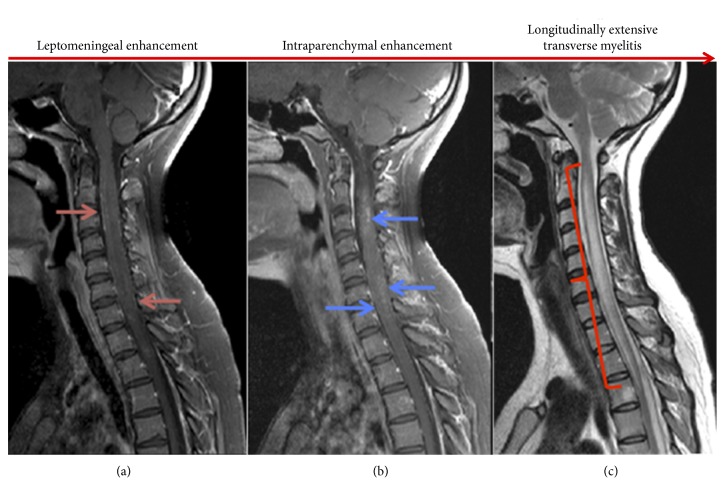
Spinal cord MRI. Sagittal gadolinium-enhanced T1-weighted images with fat saturation showing linear leptomeningeal thickening and enhancement (a) and intramedullary parenchymal patchy enhancement (b), correlating with diffuse T2-weighted hyperintense signal increase extending from C2 to Th3 (c).

A 30-year-old woman with no previous history of systemic inflammatory disease or neoplastic diseases developed loss of vision in the left eye and two days later in the right eye due to acute ON, followed by tetraparesis two weeks later. Spinal cord MRI obtained prior to treatment revealed LME, intraparenchymal contrast enhancement corresponding to the site of LME, and longitudinal extensive transverse myelitis (LETM) extending from C2 to Th3 (Figure 1).

Cerebral MRI was normal. Cerebrospinal fluid contained 122 leukocytes/mm^3^ with polymorphonuclear predominance; oligoclonal bands were not determined. Aquaporin-4 (AQP4)-IgG was negative. Accordingly, seronegative neuromyelitis optica was suspected by that time. Follow-up MRI demonstrated resolution of LME four months later.

Retrospective testing by means of two cell-based assays employing fixed and live HEK293 cells, respectively, transfected with full-length human MOG revealed the presence of MOG-IgG antibodies in a serum sample taken at onset [1, 2]. MOG-IgG seropositivity was confirmed in a second sample taken four years later.

## 3. Discussion

Inflammation in demyelinating diseases of the CNS is commonly associated with blood-brain barrier (BBB) disruption. Leptomeningeal involvement has recently been recognized as an important feature in multiple sclerosis pathogenesis [8]. LME has also been observed in AQP4-IgG-positive neuromyelitis optica spectrum disorder [9, 10]. Here, we present a case of MOG encephalomyelitis (MOG-EM) [2] with a longitudinally extensive demyelinating spinal cord lesion in which the blood-CNS barriers were disrupted, as demonstrated by gadolinium-enhanced MRI. Our findings demonstrate that spinal cord LME may occur also in MOG-EM, one of the most important differential diagnoses of MS. Notably, LME, which indicates an abnormally permeable leptomeningeal-blood barrier, was accompanying intraparenchymal BBB breakdown during an attack of acute myelitis, as visualised by contrast enhancement on T1-weighted imaging. This finding suggests that meningeal inflammation may have occurred as a bystander reaction following MOG-IgG-related parenchymal inflammation associated with subpial demyelination. Lesions involving the peripheral portions of the spinal cord indeed occur in a substantial number of cases of MOG-IgG-positive myelitis, as has recently been shown [3]. Similarly, cortical brain lesions have been described in MOG-EM, some of which were associated with LME [3, 11], and patients with MOG-IgG-positive ON may commonly present with perioptic contrast enhancement [3]. Future studies should systematically assess the presence of LME in MOG-IgG-related myelitis as well as its potential value in discriminating MOG-EM and other demyelinating diseases affecting the spinal cord.

## Glossary

AQP4: Aquaporin-4
BBB: Blood-brain barrier
CNS: Central nervous system
CSF: Cerebrospinal fluid
IgG: Immunoglobulin G
LETM: Longitudinal extensive transverse myelitis
LME: Leptomeningeal enhancement
MOG: Myelin oligodendrocyte glycoprotein
MRI: Magnetic resonance imaging
ON: Optic neuritis.
